# Exploring Disulfiram’s Anticancer Potential: PLGA Nano-Carriers for Prolonged Drug Delivery and Potential Improved Therapeutic Efficacy

**DOI:** 10.3390/nano14131133

**Published:** 2024-06-30

**Authors:** Ibrahim Dumbuya, Ana Maria Pereira, Ibrahim Tolaymat, Adnan Al Dalaty, Basel Arafat, Matt Webster, Barbara Pierscionek, Mouhamad Khoder, Mohammad Najlah

**Affiliations:** 1 Pharmaceutical Research Group, School of Allied Health, Faculty of Health, Education, Medicine and Social Care, Anglia Ruskin University, Bishops Hall Lane, Chelmsford CM1 1SQ, UK; ibrahim.dumbuya@pgr.aru.ac.uk (I.D.); ana-maria.henriques-pereira@aru.ac.uk (A.M.P.); ibrahim.tolaymat@aru.ac.uk (I.T.); adnan.al-dalaty@aru.ac.uk (A.A.D.); basel.arafat@aru.ac.uk (B.A.); barbara.pierscionek@aru.ac.uk (B.P.); 2 GMPriority Pharma Ltd., Priors Way, Coggeshall CO6 1TW, UK; 3 University of Winchester Sparkford Road, Winchester SO22 4NR, UK; matt.webster@winchester.ac.uk; 4 Faculty of Health, Science, Social Care and Education, Kingston University London, Kingston upon Thames KT1 2EE, UK; m.khoder@kingston.ac.uk

**Keywords:** breast cancer, disulfiram, nanoparticles, PLGA, direct-nanoprecipitation, PEGylated

## Abstract

Disulfiram (DS) has been shown to have potent anti-cancer activity; however, it is also characterised by its low water solubility and rapid metabolism in vivo. Biodegradable polylactic-co-glycolic acid (PLGA) polymers have been frequently employed in the manufacturing of PLGA nano-carrier drug delivery systems. Thus, to develop DS-loaded PLGA nanoparticles (NPs) capable of overcoming DS’s limitations, two methodologies were used to formulate the NPs: direct nanoprecipitation (DNP) and single emulsion/solvent evaporation (SE), followed by particle size reduction. The DNP method was demonstrated to produce NPs of superior characteristics in terms of size (151.3 nm), PDI (0.083), charge (−37.9 mV), and loading efficiency (65.3%). Consequently, NPs consisting of PLGA and encapsulated DS coated with mPEG_2k_-PLGA at adjustable ratios were prepared using the DNP method. Formulations were then characterised, and their stability in horse serum was assessed. Results revealed the PEGylated DS-loaded PLGA nano-carriers to be more efficient; hence, in-vitro studies testing these formulations were subsequently performed using two distinct breast cancer cell lines, showing great potential to significantly enhance cancer therapy.

## 1. Introduction

Despite the significant progress in cancer treatment, the incidence rate continues to rank among the highest, posing an ongoing challenge in the field of biomedical research [1]. Breast cancer (BC) is a disease characterised by high heterogeneity at both morphological and molecular levels [1], making it the most frequently diagnosed cancer among women [2] and the second leading cause of cancer-related deaths [3]. 

**Figure 1:**
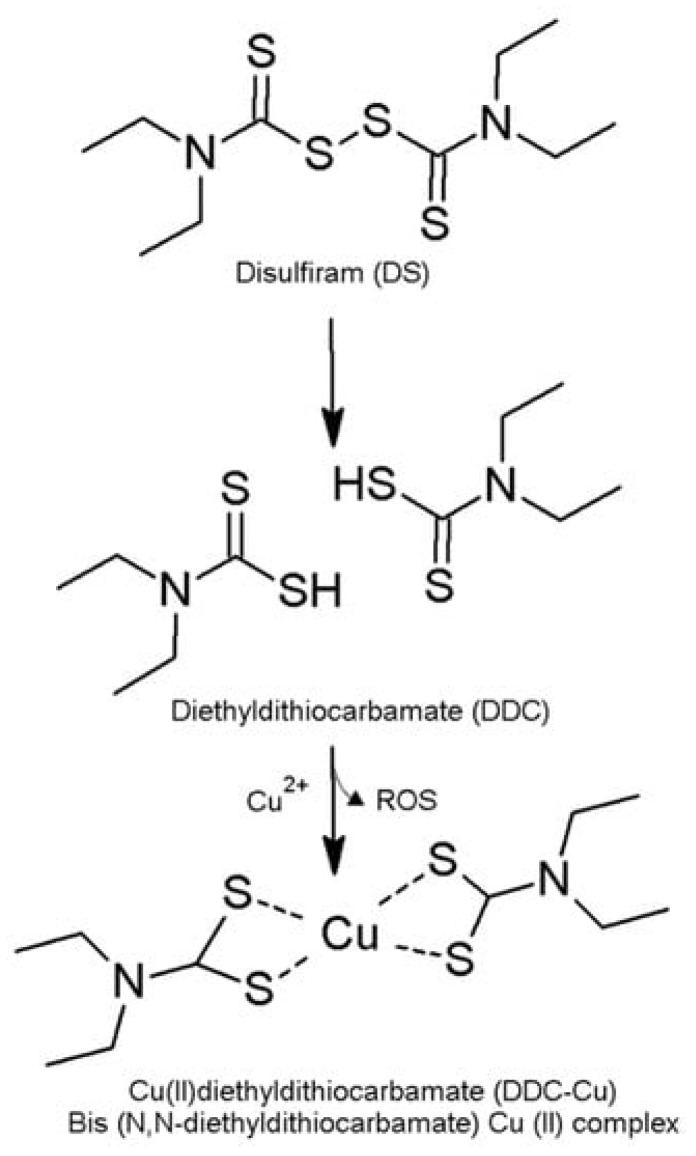
The formation of diethyldithiocarbamate copper II (DDC-Cu) through the complexation of DS with copper (Cu) [13].

The prognosis for BC is often uncertain, as it can quickly metastasise, evolving to the local lymph nodes or even the distant organs [4]. Hence, approaches like drug repurposing have been a subject of research for several years. The use of established, off-patent, non-cancer drugs with well-defined mechanisms of action presents a promising approach for acquiring cost-effective and safer therapeutic alternatives within a shortened development timeframe [5]. Disulfiram (DS), a commonly used drug to treat chronic alcoholism, has garnered significant interest for its antitumor effects when chelated with copper II to form the DDC-Cu complex (Figure 1). This complex generates reactive oxygen species (ROS), leading to cancer cell death. DS’s anticancer activity involves several mechanisms of action, including targeting aldehyde dehydrogenase, inducing the accumulation of reactive oxygen species, sensitising tumour cells to radiotherapy, suppressing DNA methylation, and overcoming drug resistance [6,7,8,9,10,11,12,13,14]. 

While DS exhibits potent anticancer effects against breast, lung, colon, prostate, ovarian, cervical, and brain cancers [14], its clinical application in cancer treatment is restricted by its rapid metabolism in the blood and poor water solubility (4.09 mg/L) [15]. Hence, further investigations to overcome these limitations are still crucial. Recent developments in nanotechnology enabled the researchers to use nanoparticles (NPs) for the purpose of enhancing the selectivity of DS-based cancer therapy, minimising degradation in the bloodstream, and improving solubility [16]. Nanoparticles can be prepared by a variety of methods, such as the emulsion-solvent evaporation (SE) method, direct nanoprecipitation (DNP), self-assembly method, etc. Most of these methods are yet to be standardised to result in efficient and accurate encapsulation efficiency and have potential challenges for scaling up. Polylactic-co-glycolic acid (PLGA), a biodegradable polymer approved by the Food and Drug Administration (FDA), has been widely used to prepare polymeric nanoparticles for drug delivery applications [17]. Wang et al. reported a significant improvement in stability and prolonged half-life (from 2 min to 7 h) when DS was loaded in PLGA NPs prepared by the SE method [18]. Moreover, coating the PLGA NPs with polyethylene glycol (PEG) was demonstrated to further improve the DS delivery performance while significantly reducing tumour size in mice tumour models [19]. Similarly, Song et al. proposed a PEGylated formulation, where the PLGA was mixed with another polymer, polycaprolactone (PCL), resulting in an improved DS encapsulation efficiency [20].

In this study, two methods of preparation of DS-loaded PLGA NPs are investigated in terms of morphology, particle size, zeta potential, encapsulation efficiency, drug release, the ability to protect DS, and the cytotoxicity against BC cell lines. Hereupon, formulations of DS-loaded PLGA NPs are developed by two different methods: DNP and SE, followed by size reduction through either probe sonication (PS) or high-pressure homogenization (HPH). In the subsequent phase, the DNP method is used to formulate PEGylated NP using variable mixed ratios of a biodegradable polymeric material, PEG-PLGA, to enhance the DS’s stability and potentially facilitate prolonged bioavailability and circulation. Additionally, we have proposed a novel method to assess unencapsulated drug content, whereas methods in the literature provide only an overall estimation. 

## 2. Materials and Methods

### 2.1. Materials

DS, PLGA 50/50 lactide/glycolide polymer (MW 19,000 Da), Poly (ethylene glycol) (PEG_2k_) methyl ether-block-poly (lactide-co-glycolide) polymer (MW 11,500 Da), Tween 80, and dimethyl sulfoxide (DMSO) were purchased from Acros Organics, Loughborough, UK. Poloxamer 188 and 3-(4, 5-dimethylthiazol-2-yl)-2, 5-diphenyl tetrazolium bromide (MTT) were purchased from Sigma Aldrich, Gillingham, UK. Sucrose, dialysis tubing (3500 MWCO), acetone, methanol, ethanol, dichloromethane (DCM), methanol (HPLC grade), pure water (HPLC grade), and phosphate-buffered saline (PBS) were obtained from Fisher Scientific, Loughborough, UK. Horse serum was purchased from Gibco, Fisher Scientific, Loughborough, UK. MDA-MB 231 and MDA-MB 231 paclitaxel (PTX) resistance cell lines were generously provided by Prof. W. Wang (The University of Wolverhampton, Wolverhampton, UK). Dulbecco’s Modified Eagle’s Medium (DMEM), fatal bovine serum (FBS), 100 units/mL of penicillin, and 100 µg/mL of streptomycin were purchased from Gibco, Fisher Scientific, Loughborough, UK. Sterilised EasYFlasks^TM^ with polystyrene filter cap, 96-well cell culture plate (flat-bottomed), and sterile petri dishes were purchased from Fisher Scientific, Loughborough, UK. All other reagents were of pharmaceutical grade and used as received.

### 2.2. Methods 

#### 2.2.1. Nanoparticle Method Development

To identify the optimal preparation method for DS-loaded PLGA NPs, a series of formulations, including both empty (i.e., DS-free) and DS-loaded PLGA NPs, were prepared using: (1) DNP without further size reduction; and (2) SE followed by particle-size reduction using either HPH or PS. For all formulations, the drug/polymer ratio was kept at 1:9 *w*/*w*.

For the DNP method, 30 mg of DS and 270 mg of polymers were dissolved in 6 mL of acetone and methanol (3:1 *v*/*v*) and heated in a water bath at 60 °C with continuous stirring at 200 rpm. Afterwards, the obtained organic phase was added dropwise to 24 mL of pre-heated (60 °C) ultra-pure water and stirred with a magnetic stirring plate (350 rpm). Then, the resulting suspension was placed under a fume cupboard for five hours, with continuous stirring, to allow the organic solvents to evaporate. Finally, the resulting formulation was centrifuged at 12,000× *g* for 5 min, and pellets were re-suspended in 2 mL of a 1% sucrose solution for lyophilization. Empty formulations were prepared using similar steps as described above, and all formulations were repeated three times.

For the SE method, the organic phase was prepared by dissolving 30 mg of DS and 270 mg of PLGA in 6 mL of dichloromethane (DCM), while the aqueous phase was made of 100 mg of polyvinyl alcohol (PVA) (surfactant) dissolved in 54 mL of ultra-pure water. The organic phase was added dropwise to the aqueous phase and left to stir for 30 min. The subsequent dispersion was subjected to size reduction using either HPH or PS techniques.

##### Particle Size Reduction by High-Pressure Homogenisation (HPH)

Formulations were subjected to four cycles using HPH (Nano DeBEE, BEE International, Billerica, MA, USA) at 20,000 psi. The resulting dispersion was left for five hours under a fume cupboard with continuous stirring at 350 rpm for the organic solvents to evaporate. The aqueous suspension was centrifuged at 12,000× *g* for 5 min and washed three times with pure water. Finally, pellets were re-suspended in sucrose (1%) for lyophilisation.

##### Particle Size Reduction by Probe Sonication (PS)

Immersed in ice, particle size reduction by PS was performed for 5 min at 60% power, following 1-min-on-and-1-min-off cycles. The resulting dispersion was left for five hours under a fume cupboard with continuous stirring at 350 rpm. The aqueous suspension was centrifuged three times for 5 min at 12,000× *g* to wash the surfactants. The pellets were prepared for lyophilisation by re-suspending in 2 mL sucrose (1%).

#### 2.2.2. Measurement of Particle Size, Polydispersity Index and Zeta Potential by Zeta Sizer

Particle size and polydispersity index (PDI) were performed by applying Photon Correlation Spectrometry using the zeta sizer Nano series (Malvern Instruments Ltd., Malvern, UK). Readings were taken three times for each measurement, and the results were expressed as mean ± SD.

#### 2.2.3. Freeze-Drying 

The resulting formulations re-suspended in sucrose (1%) were placed into a 50 mL plastic tube and sealed with parafilm, which was then pierced with a needle to allow lyophilisation. Samples were left for 2 h at −21 °C ± 0.5 °C, and then overnight at −80 °C ± 0.1 °C. Ultimately, the frozen samples were freeze-dried using LYOTAP (LTE Scientific Ltd., Greenfield, UK) for 3 days at −50 °C ± 1 °C under 0.05 ± 0.04 millibar. The final samples were kept at 4 °C to maintain their optimum conditions.

#### 2.2.4. Scanning Electron Microscopy

Scanning electron microscopy (SEM) images of the freeze-dried DS-loaded PLGA NPs were acquired using a Zeiss Evo50 electron microscope (Oxford Instrument, Abingdon, UK). Accordingly, 10 mg of each sample was weighed, suspended in 1 mL of purified water, and placed onto an SEM disc, where it was left to dry under the fume cupboard for a few hours. Lastly, the sample surface was sputter-coated with gold, and the images were taken with an accelerated voltage of 30 kV and low-vacuum conditions.

#### 2.2.5. Encapsulation Efficiency

The encapsulation efficiency (EE) of nanoparticulate formulations was calculated using the following equation:

(1) $$EE\%=\left(\frac{Total\;amount\;of\;drug-Total\;amount\;of\;free\;drug}{Total\;amount\;of\;drug}\right)\times 100\%$$

To quantify the amount of free drug (i.e., non-encapsulated drug), 10 mg of each sample was aliquoted into an Eppendorf tube containing 1 mL of methanol pre-cooled at −20 °C for 3 min. Samples were agitated for 10 s and centrifuged for 1 min at 12,000× *g*. Thereafter, the supernatant was analysed using high-performance liquid chromatography (HPLC). To calculate the amount of drug encapsulated in the NPs, pellets were dissolved in 1000 µL of DCM, vortexed for 1 min, and the supernatant was extracted and diluted in methanol for HPLC analysis. Finally, the total amount of DS in each formulation was confirmed by dissolving 10 mg in 1 mL of DCM, which was then vortexed for 1 min, diluted with methanol by the same dilution factor as the other samples, and analysed by HPLC. 

#### 2.2.6. Cumulative Release Studies

In vitro drug release studies were performed using dialysis bags to compare the cumulative release profiles of free DS and DS-loaded NPs. Cumulative percentage was calculated using the following equation:

(2) $$\%Release=\left(\frac{Total\;amount\;of\;drug\;released\;from\;the\;dialysis\;bag}{Total\;amount\;of\;drug\;encapsulated\;}\right)\times 100\%$$

For this, 10 mg of the freeze-dried sample was weighed and re-suspended in a dialysis membrane with 3 mL of PBS (10× with pH 7.4) and 1% tween 80. The dialysis bag was immersed in a dissolution of 22 mL of PBS with tween 80 (1%) and the samples were incubated in a shaking water bath at 100 rpm and 37 °C. Finally, 300 µL of the dissolution was extracted at different time intervals (4 min, 10 min, 20 min, 30 min, 1 h, 2 h, 4 h, 6 h, 8 h, and 12 h), diluted with methanol, and analysed using HPLC.

#### 2.2.7. Stability Studies in Horse Serum

The stability of free DS and the formulations were studied over a period of 8 h in horse serum. For the encapsulated DS (NPs), 5 mg of each formulation was dispersed in 5 mL of horse serum, vortexed, and incubated at 37 °C and 100 rpm. Then, 30 µL was collected at predetermined time intervals and added to 50 µL of ethanol to terminate the reaction. To crush the nanoparticles and release the encapsulated drug, 50 µL of DCM was added to the previous mixture and vortex for 1 min. Finally, 470 µL of ethanol was added, vortexed and centrifuged for 5 min at 10,000× *g*. The supernatant was then collected and analysed using HPLC.

For free DS, a reference stock of DS was prepared by dissolving 5 mg of DS in 2.5 mL of DMSO, and 25 µL was pipetted and added to 975 µL of horse serum, which was then vortexed and incubated at 37 °C and 100 rpm. At specific intervals, samples of 50 µL were added to 550 µL of ethanol to terminate the reaction. Each sample was vortexed for 1 min and centrifuged for 5 min at 12,000× *g* to separate the free DS from the denatured plasma protein. Finally, the supernatant was collected, and the resulting solution was analysed by HPLC.

To further evaluate DS stability, a linear regression of the logarithmic concentration-time plot was created for the same samples, and the elimination rate constants (*k_e_*) and half-life (*t*_1/2_) were calculated using the following equations:

(3) $$k_{e}=-\frac{slope}{2.303}$$

(4) $$t_{1/2}=-\frac{0.693}{k_{e}}$$

#### 2.2.8. HPLC Method

DS was analysed using the Ultimate High-Performance Liquid Chromatography (UHPLC) (Dionex Ultimate 3000, Thermo Scientific, Munich, Germany) following methods reported by Najlah et al. [14]. The UHPLC system is equipped with a BetaBasic C-18 4.6 mm × 150 mm (reverse phase column) of 5-µM particle size. The mobile phase consisted of water-methanol HPLC grade in a ratio of 20:80. The injection volume and flow rate used were 20 µL and 1.0 mL/min, respectively. The UV detection wavelength of DS was set at 275 nm. 

#### 2.2.9. MTT Cytotoxicity Assay

To evaluate the DS formulations cytotoxicity in vitro, two cell lines were used, the MDA-MB-231 and MDA-MB-231_PTX10_. Cells were seeded in 96-well plates at a seeding density of 10^4^ cells/well in Dulbecco’s modified Eagle’s medium (DMEM) with 10% FBS, 100 IU/mL penicillin and 100 µg/mL streptomycin. Cells were incubated overnight at 37 °C with 5% CO_2_, then exposed to a series of concentrations of freshly prepared formulations supplemented by 10 µM CuCl_2_ [21]. After 72 h, all wells were treated with a standard 3-(4, 5-dimethylthiazol-2-yl)-2, 5-diphenyltetrazolium bromide (MTT) assay. Cells treated with DS/Cu were used as positive controls. The experiments were carried out in triplicates and the percentage of cell viability and the IC50 values were calculated.

#### 2.2.10. Statistical Analysis

All statistical analysis was performed as a comparison of two groups using a paired *t*-test and a one-way analysis of variance (ANOVA) test. Post-hoc analysis was used for comparisons of the means through Turkey’s Kramer Significance Difference test. All data were expressed as the mean ± standard deviation of three separate experiments. The *p* values < 0.05 were accepted as significant.

## 3. Results and Discussion

### 3.1. Characterisation of DS-Loaded NPs Prepared by DNP and SE Methods 

#### 3.1.1. Particle Size, Polydispersity Index, Zeta Potential and Surface Morphology

**Figure 2:**
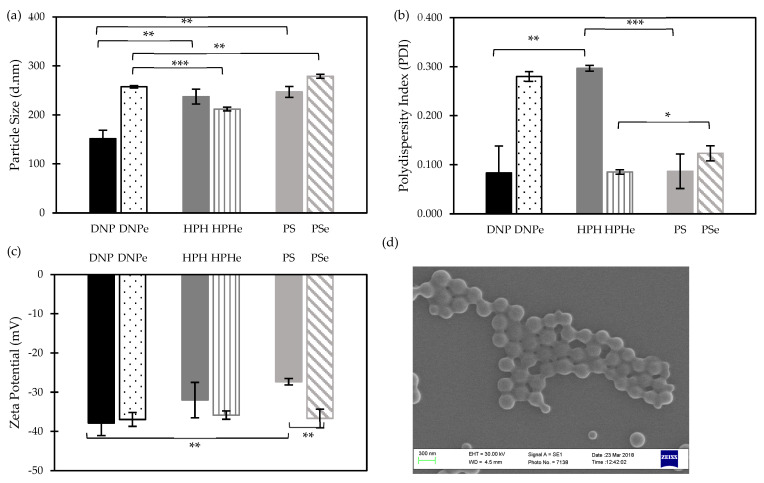
(**a**) Particle size, (**b**) polydispersity index (PDI), and (**c**) zeta potential of empty and DS-loaded PLGA nanoparticles prepared by direct nanoprecipitation (DNP) method and single emulsion/evaporation (SE) method followed by particle size reduction using high-pressure homogenization (HPH) and probe sonication (PS) (Mean ± SD, n = 3, * *p* < 0.05, ** *p* < 0.01, *** *p* < 0.001); (**d**) scanning electron microscopy (SEM), demonstrating the surface morphological images of DS-loaded PLGA NPs prepared by DNP method.

Figure 2a shows the average particle size of NP prepared by different methods. While empty NPs produced by SE followed by PS size reduction (PSe) resulted in the highest particle size (278.9 ± 4.1 nm), those prepared by the same method followed by either size reduction method were significantly smaller (*p* < 0.01) with particle sizes of 212.0 ± 2.5 nm and 257.3 ± 2.5 nm, respectively. 

However, when DS was loaded into the NP, the DNP method resulted in the smallest particles (151.3 ± 17.6 nm), which were significantly (*p* < 0.01) smaller than those prepared by SE methods (HPH 237.4 ± 15.2 and PS 246.9 ± 10.9 nm) (Figure 2a). Elnawasany et al. reported similar results with boswellic acid, curcumin, and naringin-loaded NPs, where particle size was minimal using the DNP method compared to the SE method [22].

The average PDI values of the empty and DS-loaded NPs are presented in Figure 2b. All formulations produced narrow particle size distributions (i.e., PDI < 0.3) (Figure 2b), DNPe demonstrated a significantly (*p* < 0.001) higher PDI (0.280 ± 0.010) compared to HPHe and PSe, which had a PDI of approximately 0.100. However, when DS was incorporated, unlike other formulations, the PDI of HPH increased to 0.297 ± 0.006 and became significantly (*p* < 0.01) higher than that of the DNP (0.083 ± 0.055) and PS (0.087 ± 0.035) (Figure 2b). 

Figure 2c shows the zeta potential values of nanoparticles obtained by different preparation methods. Whilst the empty formulations exhibited average zeta potential values of about—37 (*p* > 0.05) regardless of the preparation method used, the encapsulation of DS revealed some differences in zeta potential values amongst the three methods. DNP displayed significantly (*p* < 0.01) higher zeta potential compared to that of PS (Figure 2c) (−37.9 ± 3.2 and −27.3 ± 0.8, respectively). It can be argued that both homogenisation techniques, i.e., HPH and PS, apply high shear forces in comparison to DNP. This would disturb the polymeric matrix, leading to a higher amount of adsorbed drug on the surface [23,24]. Therefore, the superiority of DNP over the SE method (followed by particle size reduction) might be a consequence of the non-mechanical intervention to produce NPs [23,24]. 

Figure 2d shows the SEM image of NPs prepared by DNP, demonstrating a smooth surface, spherical shape, and well-defined structure with a homogeneously narrow particle size distribution that agrees with the PDI results discussed above.

#### 3.1.2. Encapsulation Efficiency and Cumulative Release Studies of DS-Loaded PLGA NPs

**Figure 3:**
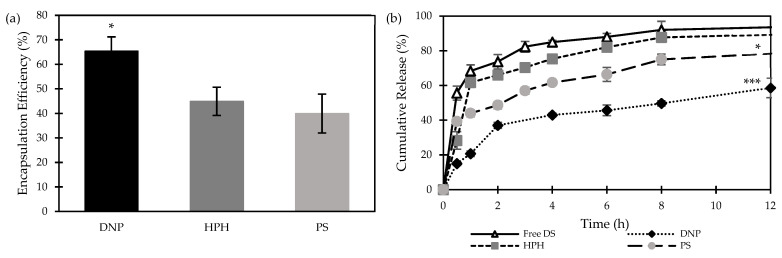
(**a**) Encapsulation efficiency of DS-loaded PLGA nanoparticles prepared by direct nanoprecipitation (DNP), single emulsion/evaporation method followed by particle size reduction using high-pressure homogenization (HPH) and probe sonication (PS) (Mean ± SD, n = 3, * *p* < 0.05, *** *p* < 0.001). (**b**) cumulative release of free DS and DS-loaded PLGA nanoparticles prepared by different nanoprecipitation methods.

Figure 3a shows the encapsulation efficiency of the DNP, PS, and HPH nanoparticles. While the DNP method was capable of achieving an EE% of 65.3 ± 5.9%, only 44.9 ± 5.8% and 39.9 ± 7.9% were achieved by SE, followed by PS and HPH, respectively (Figure 3a). This could be attributed to the outward diffusion of DS during the sonication/homogenisation stage [25]. Similar findings were reported by Elnawasany et al., confirming the superiority of the DNP method over the SE method in terms of achieving higher EE% [22].

The cumulative release profiles of free DS and DS-loaded PLGA NPs are shown in Figure 3b. As anticipated, free-DS demonstrated a significantly (*p* < 0.05) faster release profile, with around 90% diffused through the dialysis bags in 8 h and a final cumulative release of 98 ± 1.0% obtained in 24 h. For the encapsulated DS, NPs prepared by SE followed by HPH showed a comparable release profile (*p* > 0.05), suggesting the inability to sustain the drug release. Moreover, NPs prepared by DNP or SE followed by PS were more capable of delaying the drug release, as only 60% and 80%, respectively, of DS cargo were released in 12 h. 

### 3.2. Disulfiram-Loaded PLGA PEGylated Nanoparticle Formulations by Direct Nanoprecipitation Method

Having established the superiority of the DNP method in producing nanoparticles of (i) minimal particle sizes, (ii) narrow PDI, (iii) increased drug loading, and (iv) sustainable drug release, the focus of this section was to develop DS-loaded PEGylated NPs for enhanced drug biostability and potentially prolonged circulation in blood.

#### 3.2.1. Particle Size, Polydispersity Index, Zeta Potential and Surface Morphology

**Figure 4:**
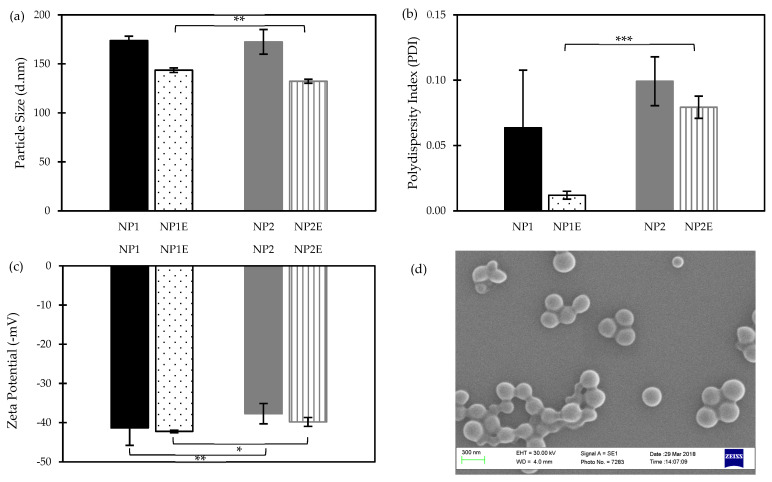
(**a**) Particle size, (**b**) polydispersity index (PDI) and (**c**) zeta potential of DS-loaded PLGA nanoparticles (NP1 and NP2) and unloaded PLGA nanoparticles (NP1E and NP2E) with a 1:9 drug/polymer ratio prepared in different organic solvents by using the DNP method (Mean ± SD, n = 3, * *p* < 0.05, ** *p* < 0.01, *** *p* < 0.001); (**d**) Scanning electron microscopy (SEM), demonstrating the surface morphological images of NP1E NP.

Table 1 shows the mixed ratios (*w*/*w*) of two DS-loaded PLGA NP formulations (NP1 to NP2) and their respective empty formulations (NP1E to NP2E). PEGylated (NP2 and NP2E) and non-PEGylated (NP1 and NP1E) PLGA NPs were prepared in a binary mixture (acetone/methanol, 3:1) with a drug-to-polymer ratio of 1:9. The results showed that the NPs average diameter was <200 nm (Figure 4a), a size smaller than the recommended suitable limit (<220 nm) for achieving passive targeting into tumour site [26].

As shown in Figure 4a, both DS-loaded NPs had a higher particle size when compared to their respective empty NPs. This could be attributed to the potential incorporation of the DS into the polymeric matrix and the consequent adsorption onto NP outer surfaces. Furthermore, a significant increase (*p* < 0.01) in particle size was observed between the empty formulations (NP1E and NP2E), possibly as a result of the PEGylation process with mPEG_2k_-PLGA. This can be attributed to the increased lateral repulsion during the addition of PEG to the lipid bilayers, which induces curvature and reduces particle size [14].

All formulations showed an acceptable size distribution (PDI < 0.1) (Figure 4b) with adequate electric surface charges (Figure 4c), with no major significance between them (*p* > 0.05). Additionally, the morphology of the different NPs was examined by SEM, which demonstrated well-defined structures, uniform spherical morphology, and smooth exterior. The particle size distribution was also found to be homogeneously narrow, confirming the minimal standard deviation of NP diameters and, respectively, low polydispersity indices of all nanoparticles (loaded and empty). Figure 4d serves as an example, showing an SEM image of NP1E.

#### 3.2.2. Encapsulation Efficiency and Cumulative Release Studies of DS-Loaded PLGA NPs

**Figure 5:**
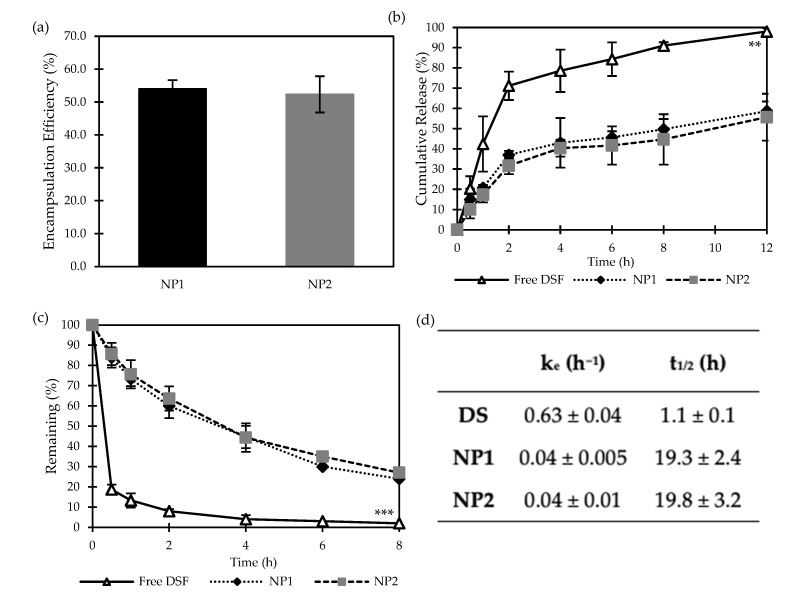
(**a**) Encapsulation efficiency of DS-loaded PLGA nanoparticles (NP1 and NP2) prepared by the DNP method (Mean ± SD, n = 3, ** *p* < 0.01, *** *p* < 0.001); (**b**) DS cumulative release and (**c**) stability in horse serum of free DS and DS-loaded PLGA nanoparticles (NP1 and NP2) prepared by the DNP method (Mean ± SD, n = 3); (**d**) Elimination rate constant (*k_e_*) and half-life (*t*_1/2_) of free DS (DS) and DS-loaded PLGA nanoparticles (NP1 and NP2) (Mean ± SD, n = 3).

The encapsulation efficiency of both loaded formulations was assessed using HPLC, and the results are represented in Figure 5a. Both formulations showed reasonable encapsulation efficiency (above 50%). It can be concluded that coating the NP exterior surfaces with mPEG_2k_-PLGA, i.e., NP2, has shown no significant effect on DS loading efficiency compared to non-PEGylated NP1.

As discussed above and shown in Figure 3b, DS-loaded PLGA NPs (drug/polymer 1:9 *w*/*w*) prepared by DNP showed high potential to maintain sustainable release profiles of DS over 24 h. Figure 5b shows the cumulative in vitro release behaviour of free DS and the NPs NP1 and NP2. It is expected that free DS (acting as the control) would release at a rate significantly (*p* < 0.05) faster than from the loaded NPs, reaching a 98 ± 1.7% cumulative release after 12 h. Conversely, NP1 and NP2 maintained a satisfactory release rate up to the 12-h study, with a percentage of the cumulative release of 58.7 ± 4.7% and 55.7 ± 11.6%, respectively. This demonstrates the capability/potential of the polymeric blends (Table 1) to significantly retain DS and minimise its outward leakage through the NP hydrophobic core-shell.

#### 3.2.3. Stability Studies in Horse Serum

It is crucial for DS to be biologically stable in the bloodstream in order to infiltrate cancer cells and induce anti-cancer effects. However, DS has an extremely short half-life (Figure 4d), hence its rapid metabolism in the bloodstream [27]. Upon ingestion, DS is converted into DDC, which, due to its hydrophilic polar nature, undergoes three possible metabolic fates: spontaneous degradation (to diethylamine and carbon disulphide), formation of glucuronide (DDC-glucuronic acid), or formation of methyl esters (DDC-Me) [28,29]. To test the performance of our NP delivery system on DS half-life, freshly prepared DS-loaded non-PEGylated (NP1) and PEGylated (NP2) nanoparticles were incubated with horse serum for 8 h in comparison to a control of free-DS (Figure 5c). As expected, free-DS was degraded completely within 30 min of incubation. On the contrary, both NP delivery systems developed in this study were proven to be effective in protecting DS instability in a physiological environment, as they significantly (*p* < 0.05) increased DS’s half-life in the serum (Figure 5d). However, as no significant difference (*p* > 0.05) in DS half-life was obtained between the PEGylated and non-PEGylated NPs, a higher mPEG_2k_-PLGA coating ratio might be required in future studies. 

#### 3.2.4. MTT Cytotoxicity Assay

**Figure 6:**
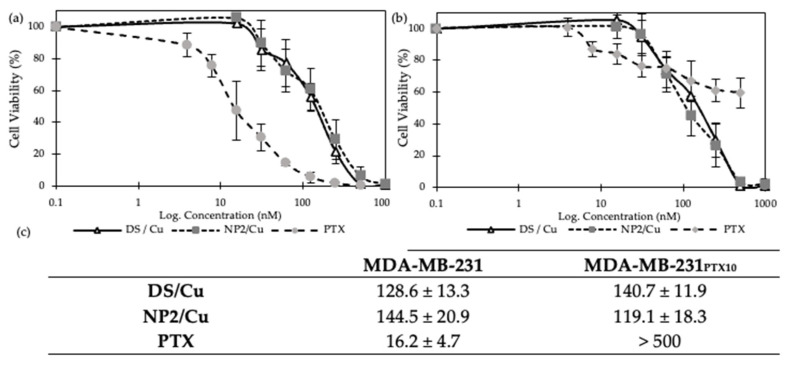
MTT assay cytotoxicity of the DS/Cu standard, paclitaxel (PTX), and DS-loaded PLGA nanoparticles (NP2/Cu) prepared by the DNP method against (**a**) MDA-MB-231 and (**b**) MDA-MB-231_PTX10_ cancer cells (Mean ± SD, n = 3); (**c**) IC_50_ values of DS/Cu, paclitaxel (PTX), and NP2/Cu against two cell lines, MDA-MB-231 and MDA-MB-231PTX10.

It has been confirmed that the efficacy of DS against cancer cells is highly enhanced by its complexation with copper [13]. The cytotoxicity effect of our DS-loaded PEGylated PLGA NPs (i.e., NP2) complexed with copper (NP2/Cu) was tested against MDA-MB-231 (Figure 6a) and MDA-MB-231_PTX10_ (Figure 6b) cell lines using the MTT assay. Accordingly, the DS/Cu complex was used as a positive control, and paclitaxel (PTX) was used to compare the resistance to PTX of both cell lines (Figure 6). Furthermore, the cytotoxicity curves of the MTT assay were deduced to determine the concentration required to inhibit cell growth by 50% (IC_50_) (Figure 6c).

When compared to the positive control, no significant difference (*p* > 0.05) in cytotoxicity effect was observed by the two formulations against either cell line, confirming that the NP is able to protect DS while maintaining the cytotoxicity of DS/Cu. The results also indicate a slow penetration rate of both formulations into PTX-resistant MDA-MB-231_PTX10_ cancer cells and non-resistant MDA-MB-231 cells. Figure 6b further demonstrates the resistance of the PTX-resistant cancer cells to paclitaxel, which is practically immune to the toxicity of the drug, contrary to the non-resistant cancer cells that are highly sensitive to PTX.

## 4. Conclusions

Disulfiram’s (DS) limited solubility has been a challenge for several years. While breakthroughs have been achieved in enhancing solubility, its effectiveness against cancer cells remains the subject of ongoing investigation by multiple researchers. PLGA-based nano-carriers have gained tremendous attention due to their numerous advantages in drug delivery. Consequently, this study has investigated the potential drug delivery modalities of DS in two phases. Firstly, DS-loaded PLGA nanoparticles (NPs) were developed using different manufacturing techniques (DNP and SE), followed by particle size reduction through either PS or HPH. DNP was superior in terms of generating smaller particle sizes, achieving a narrower polydispersity index, enhancing drug loading, and ensuring a sustained release of the drug. DS-loaded PLGA coated with PEG was successfully manufactured by the DNP method. PEGylated DS-loaded PLGA NPs exhibited increased drug entrapment, prolonged and sustainable drug release, enhanced DS stability in horse serum media, and high cytotoxicity against breast cancer cells, including those resistant to chemotherapy agents like PTX. In conclusion, DS-loaded NPs prepared by DNPs have significant potential to be used in clinical trials and within the pharmaceutical industry. Moreover, the PEGylated DS-loaded PLGA nano-carriers have great potential to significantly enhance cancer therapy; however, a higher ratio of mPEG2k-PLGA might be necessary to achieve more effective results in the future. Future work may also include in vivo studies to verify whether PEG_2k_-PLGA NPs have better stability compared to PLGA NPs. 

## Figures and Tables

**Table 1:** Ratios (*w*/*w*) of the different formulations of disulfiram (DS) loaded PLGA with PEGylated NPs (n = 3).

Formulation *	PLGA	mPEG_2k_-PLGA	DS	Total
NP1E	10	--	--	10
NP1	9	--	1	10
NP2E	9.5	0.5	--	10
NP2	8.5	0.5	1	10

* E denotes their respective empty NPs.

## Data Availability

The data presented in this study are available in this article. Enquiries may be made to the corresponding author.
