# Correction: Dumbuya et al. Exploring Disulfiram’s Anticancer Potential: PLGA Nano-Carriers for Prolonged Drug Delivery and Potential Improved Therapeutic Efficacy. *Nanomaterials* 2024, *14*, 1133

**DOI:** 10.3390/nano15211619

**Published:** 2025-10-24

**Authors:** Ibrahim Dumbuya, Ana Maria Pereira, Ibrahim Tolaymat, Adnan Al Dalaty, Basel Arafat, Matt Webster, Barbara Pierscionek, Mouhamad Khoder, Mohammad Najlah

**Affiliations:** 1 Pharmaceutical Research Group, School of Allied Health, Faculty of Health, Education, Medicine and Social Care, Anglia Ruskin University, Bishops Hall Lane, Chelmsford CM1 1SQ, UK; 2 GMPriority Pharma Ltd., Priors Way, Coggeshall CO6 1TW, UK; 3 University of Winchester Sparkford Road, Winchester SO22 4NR, UK; 4 Faculty of Health, Science, Social Care and Education, Kingston University London, Kingston upon Thames KT1 2EE, UK

In the original publication [1], the reference [1], “Kashyap, D.; Pal, D.; Sharma, R.; Garg, V.K.; Goel, N.; Koundal, D.; Zaguia, A.; Koundal, S.; Belay, A. Global Increase in Breast Cancer Incidence: Risk Factors and Preventive Measures. *BioMed Res. Int.* **2022**, *2022*, 9605439” was retracted prior to this publication, and the authors would like to replace it with the reference “Sung, H.; Ferlay, J.; Siegel, R.L.; Laversanne, M.; Soerjomataram, I.; Jemal, A.; Bray, F. Global cancer statistics 2020: GLOBOCAN estimates of incidence and mortality worldwide for 36 cancers in 185 countries. *CA Cancer J. Clin.* **2021**, *71*, 209–249”. The authors state that the scientific conclusions are unaffected. This correction was approved by the Academic Editor. The original publication has also been updated.
